# Cinnamate-Intercalated Layered Yttrium Hydroxide: UV Light-Responsive Switchable Material

**DOI:** 10.3390/mi14091791

**Published:** 2023-09-19

**Authors:** Maria A. Teplonogova, Alexey D. Yapryntsev, Alexander E. Baranchikov, Vladimir K. Ivanov

**Affiliations:** Kurnakov Institute of General and Inorganic Chemistry of the Russian Academy of Sciences, 119991 Moscow, Russia

**Keywords:** layered materials, UV-switching, anion exchange, hydrothermal treatment, clays, stimuli-responsive

## Abstract

In recent years, there has been an increasing interest in stimuli-responsive host–guest materials due to the high potential for their application in switchable devices. Light is the most convenient stimulus for operating these materials; a light-responsive guest affects the host structure and the functional characteristics of the entire material. UV-transparent layered rare earth hydroxides intercalated with UV-switchable anions are promising candidates as stimuli-responsive host–guest materials. The interlayer distance in the layered rare earth hydroxides depends on the size of the intercalated anions, which could be changed in situ, e.g., via anion isomerisation. Nevertheless, for layered rare earth hydroxides, the possibility of such changes has not been reported yet. A good candidate anion that is capable of intercalating into the interlayer space is the cinnamate anion, which undergoes UV-assisted irreversible trans–cis isomerisation. In this work, both trans- and cis-cinnamate anions were intercalated in layered yttrium hydroxide (LYH). Upon UV-irradiation, the interlayer distance of trans-cinnamate-intercalated layered yttrium hydroxide suspended in isopropanol changed from 21.9 to 20.6 Å. For the first time, the results obtained demonstrate the possibility of using layered rare earth hydroxides as stimuli-responsive materials.

## 1. Introduction

The ability to adjust material properties at the molecular level is of particular interest in the creation of switchable devices. Molecular switches are currently used in drug delivery, catalysis, molecular motors and self-assembling devices [1]. The action of external factors, such as electromagnetic, thermal, electrical, magnetic, and chemical stimuli [1,2,3], is able to change various functional characteristics of materials, including those which are structural, electronic, optical, and mechanical [1], as well as their porosity [4] and solubility [5]. For example, in a catenane-based molecular device, conductivity was shown to be sensitive to a certain voltage [6].

UV or visible irradiation are the most convenient stimuli to use with responsive materials [2]. Indeed, light is a precise and unique instrument for operating a device due to the possibility to change light wavelength or intensity, the high rate of energy transfer, and the high density of energy flux. Photosensitive (photochromic) organic molecules capable of isomerisation are the most typical components of molecular switches [7]. Using this approach, a controlled release of absorbed CO_2_ was induced via UV exposure from a metal–organic framework based on an azobenzene compound [8].

To date, light-driven changes in material structure and properties have generally been shown at a molecular level in solutions—for instance, in catenane and rotaxane molecular light-driven machines [9,10]. In a condensed state, only scarce data on light-induced transformations are available due to the rigid structure of crystalline materials. Good examples of solid-state light-responsive materials are intercalated clays and layered hydroxides [11]. These structures have a rigid layered host and a flexible organic moiety, which is capable of changing the conformation upon light irradiation. The layered structure of the hydroxides ensures relatively weak interlayer interactions and rigid intralayer bonding. This type of spatial organisation enables a change in the interlayer distance using flexible interlayer anions, maintaining the host’s basic motif [12,13].

The unique structure of layered rare earth hydroxides, consisting of rigid host layers of rare earth elements and labile anion layers, underlies the versatility of their structure-sensitive properties, such as luminescence and magnetic properties [14]. These layered rare earth hydroxide features are highly promising in theranostics and drug delivery [15,16], where light-responsive switchable materials are typically applied. Due to UV–Vis transparency [17] and anion exchange capability, layered rare earth hydroxides are convenient matrices for hosting light-sensitive anions. The important property of layered hydroxide is that its interlayer distance depends on intercalated anion size. In this way, the change in anion geometric sizes through isomerisation under UV exposure results in reversible interlayer distance changing from 20.2 to 20.3 Å in layered CoAl-hydroxide intercalated with azobenzene-4,4′-dicarboxylate [11].

The cinnamate anion exists as trans- and cis-isomers, and the transition between them occurs under UV irradiation [18]. When exposed to UV light, cinnamate changes its conformation, and this is accompanied by a change in anion length from 1.06–1.07 nm for trans-conformation [19,20,21] to 0.51–0.77 nm for cis-conformation [22]. The transition takes place through the action of UV exposure at a wavelength of about 313 nm [18]. Because of this, cinnamate is a promising candidate for the role of a UV-switchable component of a supramolecular system, which has already been confirmed—for example, for a triple-stimuli wormlike micelle based on surfactant (N-cetyl-N-methylmorpholinium bromide) and trans-cinnamic acid [23]. In this study, the structural transition, accompanied by viscosity change, of self-assembled aggregates was observed under photo-irradiation, pH, or temperature condition changes. Similarly, Saleh et al. [24] reported that, when cinnamate is encapsulated in a supramolecular system, it can lead to enhanced photoisomerisation of trans-cinnamic acid.

Cinnamate can be intercalated into layered hydroxides, as first demonstrated in an example of layered double hydroxides (LDHs) [22,25,26]. Upon UV irradiation of those LDHs, the isomerisation of the cinnamate anion occurred, according to ^13^C NMR and FT-IR analysis. Despite this, interlayer distance change was not registered. This is a surprising fact since the trans–cis isomerisation of cinnamate is accompanied by changes in anion geometric sizes. The chemical transformations of trans-cinnamate intercalated in ZnAl- and MgAl-LDHs under UV-irradiation were studied by Timar et al. [22]. According to their results, both in a solid state and in a methanol suspension, UV irradiation of trans-cinnamate in the LDHs’ matrix resulted in cinnamate isomerisation to the cis-conformation. However, according to XRD data, UV irradiation either did not affect basal spacing or led to some basal spacing expansion, which may have been due to a change in the water-to-methanol ratio in the interlayer space of LDHs. A cinnamate anion has also been successfully intercalated in a layered rare earth hydroxide, namely layered yttrium hydroxide [19]. After UV irradiation of the hybrid material, a shift of the absorption band, which is characteristic of the trans- to cis-form transition, was not observed. There were also no changes in the diffraction patterns.

The luminescent and magnetic properties of layered rare earth hydroxides depend strongly on the distance (including the interlayer space) between rare earth cations [27,28,29]. Tuning the interlayer distance in layered rare earth hydroxides using light irradiation is a promising tool for controlling their functional properties in real time. Therefore, this paper showcases the first instance of a change in the interlayer space of rare earth hydroxides driven by the UV-induced isomerisation of a photoactive ion intercalated in their structure. The work focused on the structural changes in layered yttrium hydroxide (LYH) intercalated with the cinnamate anion under UV irradiation. Under light excitation, a decrease in the interlayer distance of trans-cinnamate-intercalated layered yttrium hydroxide from 21.9 Å to 20.6 Å was demonstrated.

## 2. Materials and Methods

The following reagents were used for the syntheses without additional purification: KNO_3_ (Reachem (Moscow, Russia), chemical pure), hexamethylenetetramine (AlfaAesar (Ward Hill, MA, USA), 99+%), Y(NO_3_)_3_·6H_2_O (Lanhit, 99.99%), trans-cinnamic acid (trans-C_9_H_8_O_2_, Sigma-Aldrich (St. Louis, MO, USA), ≥99%).

The synthesis of cinnamate-intercalated layered yttrium hydroxides was performed using two methods, namely anion exchange and homogeneous hydrolysis of yttrium cinnamate.

Synthesis using the anion exchange method was performed in two stages. At the first stage, yttrium hydroxynitrate (LYH-NO_3_) was obtained through the following procedure: 26.37 g of KNO_3_ was dissolved in 300 mL of water; 5.23 g of hexamethylenetetramine was dissolved in 250 mL of water and added to a solution of potassium nitrate. The mixture obtained was added to a solution of 10.00 g Y(NO_3_)_3_·6H_2_O in 700 mL of distilled water (molar ratio Y(NO_3_)_3_:hexamethylenetetramine:KNO_3_ = 1:1.43:10). The volume of the final solution was adjusted to 2 L with distilled water.

The precursor solution was placed in a round-bottom flask and heated with a reflux condenser under vigorous stirring at 90 °C for 40 min. During the synthesis, the formation of a white suspension was observed. After cooling, the suspension was filtered through a glass filter (POR4) and the sediment was washed with distilled water and dried in a desiccator at 50 °C and 75% relative humidity.

Synthesis via anion exchange reaction was performed as follows. Since layered REE hydroxides are stable in a narrow pH range only (about 5.5–7.5), cinnamic acid was first converted to potassium salt by adjusting the pH of the solutions to 6.8–7.0 with diluted potassium hydroxide solution. To the solution obtained, a powder of yttrium hydroxynitrate (0.05–0.1 g) at a ratio of 1 mol Y_2_(OH)_5_NO_3_·1.5H_2_O per 3 mol of cinnamic acid was added. The suspension was transferred to a Teflon autoclave (50% filling degree) and subjected to hydrothermal treatment at 120–160 °C for 24 h. Exchange reactions were also carried out at 25 °C for 24 h, with constant stirring of the suspensions, to determine the effect of the temperature of the anion exchange on the composition of the products. After synthesis, the precipitates were separated on a glass filter (POR4), washed with distilled water, and dried at 50 °C in a desiccator.

For homogeneous hydrolysis synthesis, a solution containing potassium cinnamate (pH~6.8–7.0) was added to a 0.1 M solution of Y(NO_3_)_3_, under stirring, at the ratio Y^3+^:cinnamate = 1:1.5. Upon mixing the solutions of yttrium nitrate and organic acids, a white precipitate of yttrium cinnamate was formed. The precipitate was filtered on a glass filter, dried at 50 °C, and studied using X-ray diffraction. Subsequently, the powder obtained was suspended in 45 mL of distilled water, a hexamethylenetetramine solution (0.2 g in 5 mL of distilled water) was added to the suspension, and the resulting mixture was subjected to hydrothermal treatment at 120 °C. The precipitate was then filtered on a glass filter (POR4), washed with distilled water, and dried at 50 °C in the desiccator.

To convert the trans form of cinnamate to the cis form, the solution of potassium trans-cinnamate was irradiated with an UV lamp with an emission wavelength of 312 nm at 65 W power. UV irradiation of trans-cinnamate-intercalated LYH powders was performed for 48 h using a 12 W lamp at a wavelength of 312 nm. A suspension of trans-cinnamate-intercalated LYH (~0.4 g) in 50 mL of isopropanol was irradiated with 312 nm UV (12 W) under constant stirring. At fixed time intervals (45 min and 1.5, 4, 7, 24, 28, 31, 48 h, and 52.5 h), 5 mL aliquots of the suspension were taken, and the powder was separated, washed, and dried at 50 °C for the further X-ray diffraction studies. The volume of the isopropanol suspension was kept constant by adding the corresponding portions of isopropanol.

X-ray diffraction (XRD) analysis of the samples was performed on a Bruker (Billerica, MA, USA) D8 Advance diffractometer (CuKα-radiation, Ni filter) in the Bragg–Brentano geometry using LYNXeye detector. All diffraction patterns were registered in the range of 2.5–55° 2 θ, in steps of 0.02° 2 θ and with an exposure time of not less than 0.05 sec/point. Diffraction patterns were indexed using the PDF2 database (2012). From the position on the 2 θ axis of the first reflection of the series, the basal interlayer distance of the layered phases obtained could be calculated from the Bragg–Wullf condition: d = λ/2 sin θ (λ = 1.54051 Å for the copper cathode used). A full-profile refinement was carried out using TOPAS (v. 4.2) software.

Raman spectra were recorded using a Renishaw InVia (Great Britain) multichannel spectrometer with a 514 nm argon laser or a 633 nm helium–neon laser as an emission source. Measurements were made in the backscattering geometry using a Leica DMLM confocal microscope at room temperature in air. A laser beam was focused on the sample using an optical microscope objective (magnification ×50). Spectra were recorded in the range 100–4000 cm^−1^, in 1 cm^−1^ increments, (spectra accumulation was varied from 10 to 100 s). 

IR spectra of the powders were recorded on a Bruker ALPHA device (diamond attachment) in the range 400–4000 cm^−1^ in the mode of attenuated total reflectance.

The Vesta (v. 3.5.7) software was used to draw the scheme of the layered yttrium hydroxide crystal structure.

## 3. Results and Discussion

### 3.1. Synthesis of Layered Yttrium Hydroxides Intercalated with Trans-Cinnamate Anions

**Figure 1:**
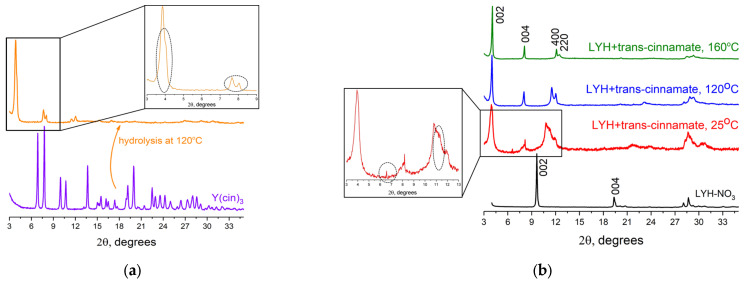
X-ray diffraction patterns of the trans-cinnamate-intercalated layered yttrium hydroxide (**a**) using the hydrolysis of yttrium cinnamate Y(cin)_3_ and (**b**) the ion exchange method using yttrium hydroxynitrate LYH-NO_3_ and potassium trans-cinnamate.

Initially, an attempt was made to intercalate trans-cinnamate into LYH via the hydrolysis of yttrium cinnamate [30,31]. Yttrium cinnamate Y(C_8_H_7_COO)_3_ was obtained by mixing potassium cinnamate and yttrium nitrate solutions at room temperature. According to XRD results (Figure 1a), the diffraction pattern of this solid product (Y(cin)_3_) coincides with data reported previously for yttrium cinnamate [32]. Subsequent hydrothermal treatment of Y(C_8_H_7_COO)_3_ in the presence of hexamethylenetetramine resulted in the formation of a multi-phase product (Figure 1a, inset). This is indicated by the reflex at ~8° 2 θ in addition to the reflexes of the 00*l* series characteristic of layered hydroxides and the shoulder of this peak at ~4° 2 θ. Most probably, this impurity corresponds with the phase of layered yttrium hydroxide with another hydrate composition. The presence of several different hydrate phases is typical for layered rare earth hydroxides; interlayer distance in these phases can differ by 0.2–0.3 Å [14].

For the synthesis of trans-cinnamate-intercalated layered yttrium hydroxide via the anion exchange method, layered yttrium hydroxynitrate (LYH-NO_3_) was synthesised through homogeneous hydrolysis of yttrium nitrate [33]. According to X-ray diffraction data, the diffraction pattern of the solid-phase product (LYH-NO_3_ in Figure 1b) coincides with the reported recently diffraction pattern of layered yttrium hydroxynitrate Y_2_(OH)_5_NO_3_·nH_2_O [34]. The layered yttrium hydroxynitrate crystallises in the monoclinic crystal system [35], with the unit cell parameters a = 13.229 ± 0.001, b = 7.010 ± 0.002, and c = 9.162 ± 0.001 Å (space group *P*2_1_; see Supplementary Materials, Figure S1 and Table S1). In general, a characteristic feature of the diffraction patterns of layered hydroxides was the presence of 00*l* series reflexes, with the parameter c of the unit cell corresponding to the basal interlayer distance. Subtracting the thickness of the metal hydroxide layer of layered yttrium hydroxide (~5.5–6.5 Å, according to calculations based on crystallographic data [36]) from the basal distance, the size of the interlayer space could be calculated. In the case of layered yttrium hydroxynitrate, it was ~4.3 Å.

After the anion exchange between yttrium hydroxynitrate and potassium trans-cinnamate at different temperatures, the layered phases showing a set of multiple 00*l*-series reflections were obtained (Figure 1b). At 25 °C, a layered phase with a basal interlayer distance of ~22.2 Å was formed (Figure 1b). The increase in the interlayer distance of the layered yttrium hydroxide compared with the layered hydroxynitrate (~9.8 Å) was related to the replacement of nitrate ions by larger trans-cinnamate anions. At the same time, broad lines in the diffraction pattern of trans-cinnamate-intercalated LYH, obtained at 25 °C, indicated a low level of crystallinity of the sample; thus, it was supposed that the increase in the temperature of anion exchange reaction would contribute to higher crystallinity of the LYH due to the Ostwald ripening. Moreover, in the diffraction pattern of the sample obtained at 25 °C, the peaks at 6.5 and 11.5° 2 θ indicate the presence of some impurities (see Figure 1b, inset). The authors were unable to attribute these peaks; however, they do not correspond to carbonate phases (Y_2_(CO_3_)_3_·nH_2_O, PDF2 cards ## 24–1419, 30–1444, 70–278, 81–1538), being the most typical admixtures in LYHs. As was supposed, the rise in temperature to 120–160 °C made it possible to synthesise a well-crystallised layered phase with no admixtures (Figure 1b). Thus, for the synthesis of trans-cinnamate-intercalated LYH using the ion exchange method, elevated temperatures are preferred. This observation is in line with the results reported elsewhere [37,38]. The products of anion exchange at 120–160 °C possessed a basal interlayer distance of about 21.7 Å. By subtracting from this value the thickness of the metal hydroxide layer of layered yttrium hydroxide (5–6 Å [36]), the size of the interlayer space between the layers could be determined; this was equal to ~16 Å, thus exceeding the size of the trans-cinnamate anion (10.6–10.7 Å [19,20,21]) and indicating its bilayer packing in the interlayer space of layered yttrium hydroxide with possible partial overlapping of the anion layers. The results obtained are consistent with previously published reports (see, e.g., [19]), where the basal interlayer distance in the layered yttrium hydroxide intercalated with trans-cinnamate was found to be 20.0 Å, indicating the bilayer packing of anions with partial interpenetration of the anion layers. Another possible explanation could be the tilted orientation of the anions relative to the metal hydroxide layers, as assumed by Li et al., for Zn_2_Ti-LDHs intercalated with trans-cinnamate [21].

Thus, according to XRD data, intercalation of the trans form of cinnamate to produce a single-phase product was successfully realised only through ion exchange under hydrothermal conditions at 120–160 °C.

**Figure 2:**
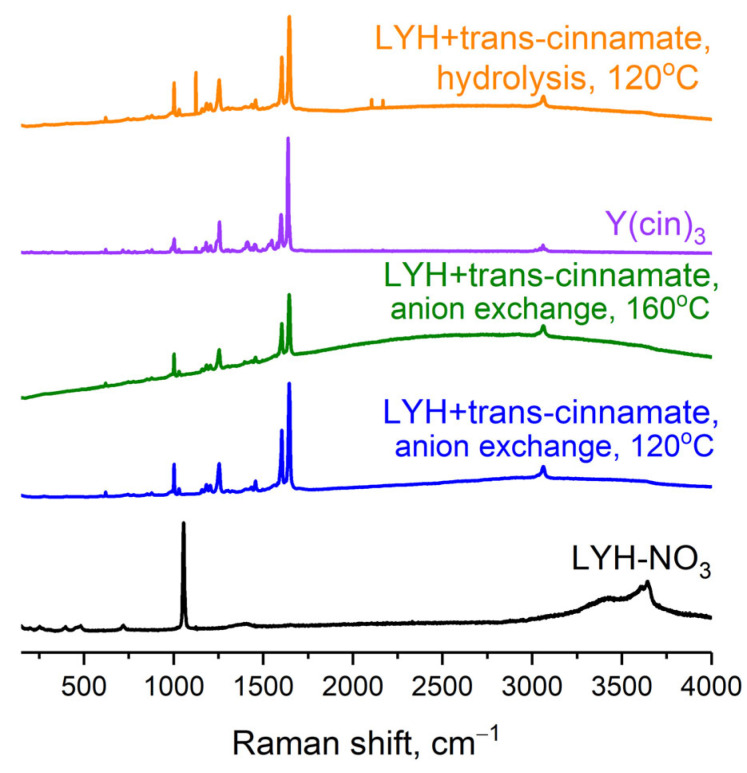
Raman spectra of LYH-NO_3_ and trans-cinnamate-intercalated LYH samples synthesised via anion exchange or by yttrium cinnamate homogeneous hydrolysis methods; yttrium cinnamate Y(cin)_3_.

Since the thermal decomposition of cinnamic acid begins at 140–155 °C [39], the samples obtained were studied using Raman spectroscopy to confirm their thermal stability and, additionally, to establish whether anion exchange was complete. The Raman spectroscopic data confirmed the successful intercalation of trans-cinnamate into the layered yttrium hydroxide matrix by ion exchange and homogeneous hydrolysis methods at 120 °C and even at 160 °C (Figure 2). Comparison with existing data (see Supplementary Materials, Table S2) [40] demonstrated that the spectra of the obtained compounds showed bands characteristic of cinnamic acid derivatives: the bands at 620 cm^−1^ corresponded to vibrations of the benzene ring, the bands at 850–880 cm^−1^ corresponded to vibrations of carboxylate groups, the bands at 1000–1300 cm^−1^ corresponded to the deformation vibrations of C-H bonds of aliphatic and aromatic fragments, and the bands at 1450–1500 cm^−1^ corresponded to C-H deformation vibrations of double bonds. At 1640 cm^−1^, there was an intense peak corresponding to the presence of a carbon–carbon double bond.

The Raman spectra of layered yttrium hydroxide intercalated with trans-cinnamate using different methods lacked bands at 719 cm^−1^ and 1054 cm^−1^ (Figure 2), corresponding to vibrations of the nitrate anion [41]. The absence of NO_3_^−^-vibration bands confirmed the completeness of ion exchange in the interlayer space of the layered hydroxide. Thus, all further synthesis of trans-cinnamate-intercalated LYH was carried out by the anion exchange method under hydrothermal conditions (120 °C).

### 3.2. Synthesis of Layered Yttrium Hydroxides Intercalated with Cis-Cinnamate Anions

The next part of the research was focused on the synthesis of cis-cinnamate and its intercalation into LYH, taking into account the fact that, to the best of the authors’ knowledge, there are currently no papers devoted to cis-cinnamate-intercalated layered rare earth hydroxides.

**Figure 3:**
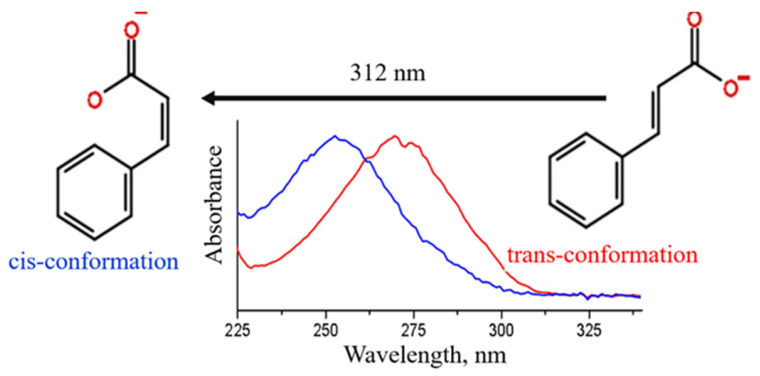
UV–Vis spectra of trans- and cis-cinnamate anions used in the present study.

To conversion the trans form of cinnamate ion to the cis form was performed via UV irradiation of the potassium trans-cinnamate aqueous solution. The UV–visible spectrum of the UV-irradiated potassium cinnamate (Figure 3) corresponds to the spectrum of cis-cinnamate [22]. It should be noted that the transition from the trans form to the cis form does not usually occur completely: the conversion degree is typically 3–80% [42]. A complete transition to cis-cinnamate can only be achieved by using special solvents such as ionic liquids [43]. The cis form is quite stable and can be converted to the trans form with high yield only under special conditions using tetrahydrofuran as a solvent and I_2_ as a catalyst [42]; in this work, the reverse transition from the cis to the trans form was not performed.

**Figure 4:**
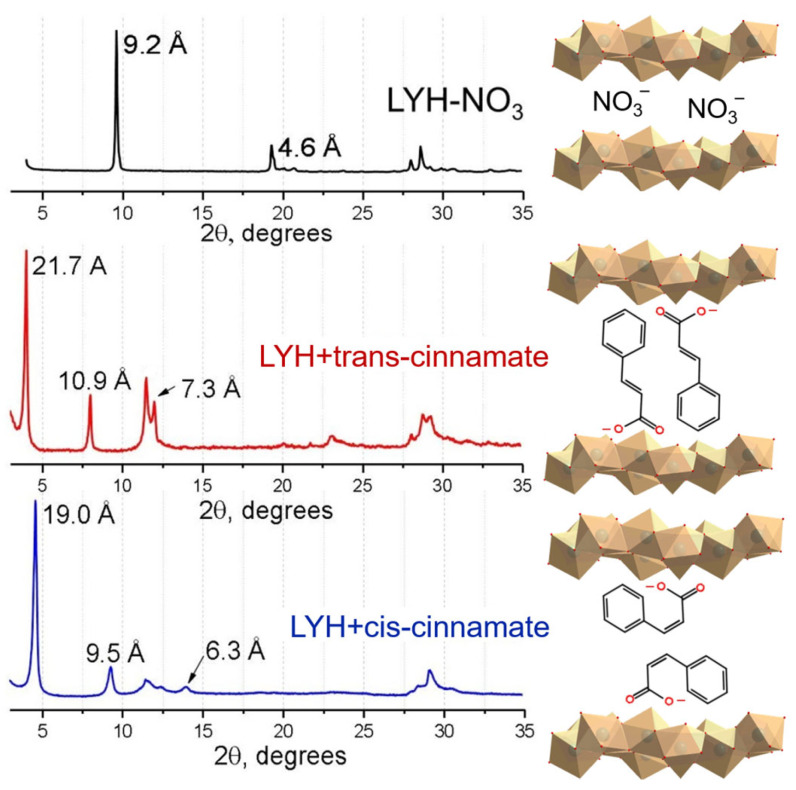
Diffraction patterns and supposed structures of LYH-NO_3_ and the products of its interaction under hydrothermal conditions at 120 °C with aqueous solutions of trans-cinnamate and cis-cinnamate.

Intercalation of the as-synthesised cis-cinnamate was also performed using anion exchange method under hydrothermal conditions at 120 °C. The diffraction patterns of the products of trans- and cis-isomers’ intercalation in the layered yttrium hydroxide (trans-cinnamate-intercalated LYH, LYH-trans) and cis-cinnamate-intercalated LYH, LYH-cis), respectively) differed in the position of (00*l*) reflexes (Figure 4). The basal interlayer distance in trans-cinnamate-intercalated LYH (21.7 Å) was greater than the basal distance in cis-cinnamate-intercalated LYH (19.0 Å). This fact can be explained by the larger size of the trans-cinnamate anion (10.6–10.7 Å) compared to the cis-cinnamate anion (5.1–7.7 Å [22]). Note that in the case of the cis-conformation, the bilayer packing of anions was probably also realised (Figure 4) since the thickness of the metal hydroxide layer was 5–6 Å and, accordingly, the interlayer space was ~14.5 Å, which was approximately twice the size of the cis-anion. It is most likely that bilayer packing in the case of cis-cinnamate-intercalated LYH occurred without interdigitating the anion layers. In contrast, in trans-cinnamate-intercalated LYH, there was an overlapping of the trans-cinnamate layers. This assumption is consistent with data on cis-cinnamate intercalation in Mg_2_Al- and Zn_2_Al-LDHs [22].

**Figure 5:**
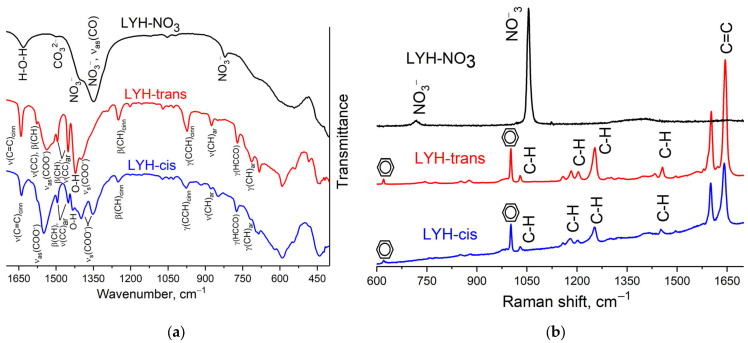
(**a**) IR- and (**b**) Raman spectra of LYH-NO_3_, trans-cinnamate-intercalated LYH (LYH-trans) and cis-cinnamate-intercalated LYH (LYH-cis).

To control the completeness of anion exchange and to detect the presence of cinnamate anion in the products, Raman spectroscopy studies were performed. As shown in Figure 5b, the Raman spectra of trans-cinnamate-intercalated LYH and cis-cinnamate-intercalated LYH are virtually indistinguishable, which is consistent with the literature [44]. In the Raman spectra obtained, the bands corresponding to vibrations of C=C bonds (1644 cm^−1^) and C-H bonds (1497, 1456, 1253, 1203, 1182, 1158 and 1030 cm^−1^), as well as vibrations of the aromatic ring (1603 and 1002 cm^−1^), are well expressed, confirming the presence of cinnamate in the layered yttrium hydroxide’s composition. The detailed assignment of the bands to their corresponding vibrations is given in Table S2 of the Supplementary Materials. Note that in the Raman spectra of trans-cinnamate- and cis-cinnamate-intercalated LYHs, there are no bands corresponding to the vibrations of the NO_3_^−^-group (1406, 1055 and 719 cm^−1^), indicating that ion exchange was complete.

The IR spectrum of the LYH-NO_3_ sample shows intensive bands which are characteristic of layered rare earth hydroxynitrates (Figure 5a) [34,45]. The band at 1633 cm^−1^ corresponds to bending ν_2_ mode of H–O–H vibrations [36]. Several broadened bands in the region of 1350–1410 cm^−1^ are characteristic of nitrate ions in the structure of layered rare earth hydroxides [46,47,48,49,50]. The exact attribution of these bands is complicated due to the structural disorder inherent in rare earth hydroxynitrates; the band at 1402 cm^−1^ relates to the ν_4_ asymmetric stretch O–NO_2_ vibrations [36], and the band at 1348 cm^−1^ can be assigned to the ν_3_ vibration of a non-coordinated nitrate group (D_3h_) in the interlayer space [45,51]. The band at 821 cm^−1^ is due to the ν_7_ mode of NO_3_^−^ vibrations [46].

The IR spectra of the trans-cinnamate and cis-cinnamate-intercalated LYHs (Figure 5a) contain intense bands corresponding to the vibrations of the C=C bond (~1640 cm^−1^), the O-H group in the bond plane (~1420 cm^−1^), and asymmetric and symmetric vibrations of the COO-group (~1550 and 1400 cm^−1^, respectively), which is in agreement with data on the spectra of LDHs intercalated with cinnamate [21,22]. In general, comparison of the recently reported IR spectra with the IR spectrum of the precursor trans-cinnamic acid (see Supplementary Materials, Table S3; [22]) enabled confirmation that, in the trans-cinnamate-intercalated LYH sample, the anion was exactly in the trans form (with characteristic vibration bands at ~1578, 1398, 1292, and 1250 cm^−1^). In the IR spectrum of cis-cinnamate-intercalated LYH (Figure 5a), few bands characteristic of the cis form of the anion (1555 and 1356 cm^−1^) appeared; these bands are absent in the IR spectrum of trans-cinnamate-intercalated LYH (see Supplementary Materials, Table S3; [22]). However, several bands corresponding to trans-cinnamate vibrations appeared in the IR spectrum of the cis-cinnamate-intercalated LYH (1577, 1292 and 1249 cm^−1^). The preservation of the trans-cinnamate bands proves the incomplete transition of the trans- to cis-conformation during UV irradiation of the potassium cinnamate solution before intercalation, which agrees with existing data on the incomplete trans–cis transition under normal conditions [43]. However, XRD data showed significant differences between trans-cinnamate-intercalated LYH and cis-cinnamate-intercalated LYH diffraction patterns, mainly in the positions of 00*l* series peaks (see Figure 4). The cis-cinnamate-intercalated LYH XRD pattern does not possess a peak at 4° corresponding to the 21.7 Å basal interlayer distance in trans-cinnamate-intercalated LYH. On the basis of this fact, the authors assume that the trans-cinnamate anion is contained mostly on the cis-cinnamate-intercalated LYH surface. In the mixed solution of cis- and trans-cinnamate, the cis form is expected to penetrate into the interlayer space of LYH easier than the trans-form, since the intercalation of trans-cinnamate is hindered due to the smaller interlayer distance in the cis-intercalated LYH. Thus, the trans-anion remains in the mother liquor and could be absorbed on the cis-cinnamate-intercalated LYH surface.

### 3.3. UV-Induced Transformation of Trans-Cinnamate-Intercalated LYH

After UV-irradiation, the cinnamate anion isomerises, which leads to a change in the anion’s geometric size. When cinnamate is intercalated in the layered hydroxide, the change in the anion size will probably induce the corresponding change in the hydroxide interlayer distance. Although such changes were not observed in the cinnamate-intercalated LDHs [22], layered rare earth hydroxides are probably better matrices, for the following reasons. Firstly, layered rare earth hydroxide surface charge density is higher than in LDHs [14]; consequently, the density of intercalated anions will be higher in layered rare earth hydroxides than in LDHs. Secondly, in the layered rare earth hydroxide gallery, water molecules are directly coordinated to rare earth ions; conversely, in the LDHs, water is located freely in the interlayer space [36]. Thus, in layered rare earth hydroxides, hydrogen bonds between the host layers should be weaker than in LDHs. Taking into account the higher anion density and the weaker hydrogen bonds, it can be supposed that interlayer distance change in cinnamate-intercalated layered rare earth hydroxides can be activated more easily than in LDHs.

**Figure 6:**
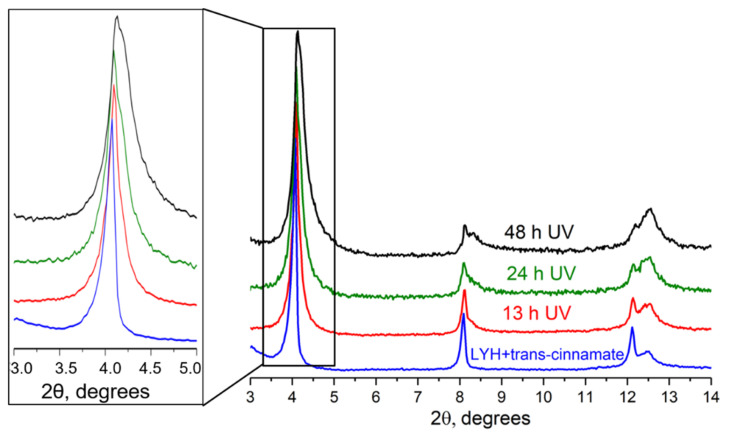
Diffraction patterns of the initial trans-cinnamate-intercalated LYH and products of UV exposure of trans-cinnamate-intercalated LYH powders, for 13, 24, and 48 h.

In order to experimentally confirm this hypothesis, the powders of layered yttrium hydroxides intercalated with the trans form of cinnamate ion were UV-irradiated for different periods of time. Figure 6 shows the powder diffraction patterns recorded after each ~12 h of exposure. According to a full-profile analysis, the *c* parameter values were 22.00 ± 0.01 Å, 21.96 ± 0.01 Å, 21.83 ± 0.03 Å and 21.45 ± 0.02 Å for 0, 13, 24, and 48 h of exposure to UV radiation, respectively, indicating that a mere ~0.5 Å decrease in the basal interlayer distance occurred upon exposure of the powder. Longer UV exposure (up to seven days) did not additionally affect the basal interlayer distance (Figure 6). It is worth noting that Kim et al. [19] observed no changes in the crystal structure of cinnamate-intercalated LYH after 24 h of the UV irradiation of the sample in the form of a powder. Data derived from the current study, however, indicated that, after UV irradiation, a slight broadening of diffraction lines occurred: a full width at half maximum β_001_ changed from 0.11° to 0.32° (Figure 6), indicating possible microstrains between metal hydroxide layers. These microstrains might have appeared due to local trans- to cis-cinnamate transitions causing distortions in the layered structure.

Thus, the crystal structure of trans-cinnamate-intercalated LYH did not change after 48 h of UV irradiation of the powder. This result brought us to search for the other ways to facilitate the cinnamate isomerisation in the interlayer space of LYH.

According to Timar et al. [22], the low flexibility of the hydroxide hosts is due to the hydrogen bonding of the layers. Timar et al., proposed the weakening of H-bonding in trans-cinnamate-intercalated Mg_2_Al-LDH through water-to-methanol replacement. In the present study, it was decided to follow this approach by subjecting the isopropanol suspension of trans-cinnamate-intercalated LYH powder to UV irradiation.

**Figure 7:**
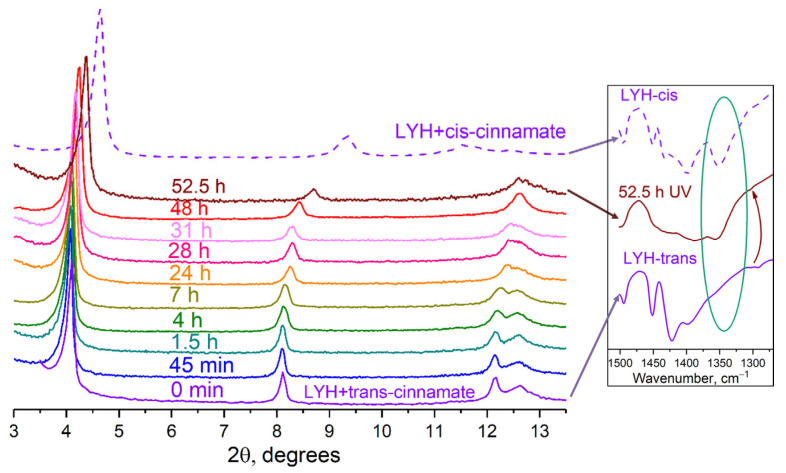
(**Left panel**) The diffraction patterns of the trans-cinnamate-intercalated LYH before and after UV irradiation of the isopropanol suspension of trans-cinnamate-intercalated LYH at different time intervals; a diffraction pattern of cis-cinnamate-intercalated LYH is also shown. (**Right panel**) Fragments of the IR spectra of the selected samples.

Figure 7 shows the diffraction patterns recorded after UV irradiation of trans-cinnamate-intercalated LYH isopropanol suspensions. For these diffraction patterns, a full-profile fitting was performed, and the parameters of the unit cell were determined. It was found that in the process of irradiation, reflexes of 00*l* series shifted to the larger angles. It is most probable that this was due to the gradual transformation of the trans-cinnamate into the cis form in the interlayer space of layered yttrium hydroxide. It should be emphasised that no significant amorphisation was observed in these experiments. The full width at half maximum β_001_ varied from 0.08° to 0.15°, indicating fewer microstrains than with LYH powder after UV irradiation (see above).

**Figure 8:**
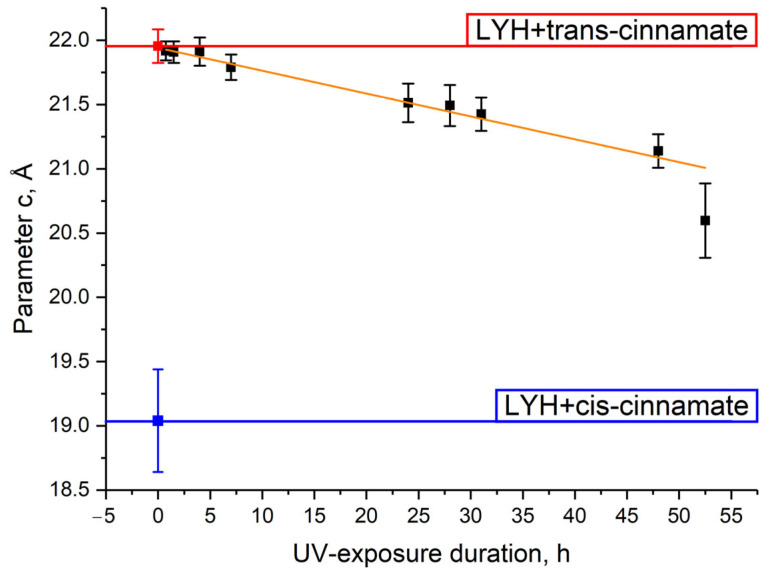
Dependence of the interlayer basal distance (unit cell parameter *c*) of trans-cinnamate-intercalated LYH on the duration of UV exposure.

The basal interlayer distance for trans-cinnamate-intercalated LYH changed from 21.96 ± 0.01 to 20.60 ± 0.03 Å after 52.5 h of UV irradiation (Figure 8; see full refinement results in Supplementary Materials, Figure S1 and Table S1).

The maximum value of the basal interlayer distance for UV-irradiated trans-cinnamate-intercalated LYH samples still did not achieve a value characteristic of cis-cinnamate-intercalated LYH, implying an incomplete transition from trans- to cis-cinnamate (see Figure 7 and Figure 8). This result is in agreement with data presented elsewhere for cinnamate-intercalated LDHs [22]. The reasons for this are as follows. Firstly, in the interlayer space of layered rare earth hydroxides, the confinement effect could have affected the intercalated species [52,53,54,55]. The isomerisation of cinnamate would also be hindered under these conditions. Secondly, the host layers are extended and hard-to-move structures, in comparison with isomerising anions. Thirdly, UV rays are partly scattered on the layered hydroxide matrix [56], which reduces the efficiency of irradiation. Nevertheless, the interlayer distance change in the trans-cinnamate-intercalated LYH sample during UV exposure of the isopropanol suspension was clearly evident.

IR spectrum of the LYH sample after UV irradiation for 52.5 h shows the band at ~1350 cm^−1^ indicating the presence of cis-cinnamate in the layered yttrium hydroxide and supporting the cinnamate isomerisation upon UV irradiation (see inset in Figure 7).

One can assume that the interlayer distance in LYH could be changed by the deintercalation of the cinnamate-anion into isopropanol. Such an effect was previously observed with the aqueous suspensions of LDHs, where the cinnamate anion was replaced by CO_3_^2−^ [57], but LYH has been shown to be significantly more stable against cinnamate anion deintercalation [19]. Alternatively, the changes in the interlayer space could be also due to the changes in the amount of water molecules incorporated between the oxide layers [14]. In order to prove the negligible effect of cinnamate deintercalation or the change in water content on the interlayer distance, a control experiment was performed. Trans-cinnamate-intercalated LYH powder was suspended in isopropanol and stirred for 48 h in the dark. Indeed, trans-cinnamate deintercalation occurred under these conditions, as proved by mother liquor absorbance spectra (see Supplementary Materials, Figure S2). The spectrum obtained corresponded to trans-cinnamate spectra with an absorption maximum at 270 nm. According to the authors’ estimates, the amount of deintercalated cinnamate did not exceed 4–10 mol%. However, upon such treatment, no shift in 00*l* peaks’ position in the diffraction pattern of the resulting product was registered (see Supplementary Materials, Figure S3). Note that in the trans-cinnamate-intercalated LYH suspensions, there were no extraneous anions (organic, nitrate, chloride, etc.). This means that the only phase that could form in these suspensions was layered yttrium hydroxycarbonate, although the presence of this phase was not confirmed by analysis of the corresponding diffraction patterns, (additional 00*l* peaks were not observed). According to the results of full-profile analysis, there were also no changes in the unit cell parameter of the layered yttrium hydroxide intercalated by trans-cinnamate ions upon soaking in isopropanol for 48 h in the dark (21.95 ± 0.02 Å for the starting sample and 21.97 ± 0.01 Å for the final sample). Thus, it can be concluded that the ion exchange of cinnamate for carbonate or the changes in water content was negligibly low and that the interlayer distance change occurred as a result of the UV isomerisation of cinnamate intercalated in layered yttrium hydroxide.

## 4. Conclusions

In this study, light-response changes in the crystal structure of a layered rare earth hydroxide intercalated with an organic ion capable of cis–trans isomerisation were demonstrated. After UV irradiation of the powder of layered yttrium hydroxide intercalated with trans-cinnamate, the interlayer distance decreased from 22.0 Å to 21.5 Å. For the isopropanol suspension of this compound, an almost linear decrease of interlayer distance from 21.9 Å to 20.6 Å with irradiation time was registered. The results obtained provide an approach for the further design of solid-state supramolecular actuators based on layered rare earth hydroxides and operated by light. The structural changes can be considered as an efficient way of tuning structure-sensitive properties, including the luminescence and magnetic properties of layered rare earth hydroxides.

## Data Availability

The data generated in the present study are available from the corresponding author upon reasonable request.

## Supplementary Materials

The following supporting information can be downloaded at: https://www.mdpi.com/article/10.3390/mi14091791/s1, Table S1: the results of full-profile refinement for LYH-NO3, trans-cinnamate (LYH-trans), and cis-cinnamate (LYH-cis)-intercalated LYH sample (space group P21); Table S2: position of the bands (cm^−1^) in the Raman spectra of layered yttrium hydroxide (LYH) intercalated with cinnamate and assignment of the vibrations according to literature data; Table S3: position of the bands (cm^−1^) in the infrared spectra of layered yttrium hydroxide (LYH) intercalated with cinnamate and assignment of the vibrations according to literature data; Figure S1: the full-profile refinement charts for the samples of (a) LYH-NO3; (b) trans-cinnamate-intercalated LYH (LYH-trans); (c) cis-cinnamate-intercalated LYH (LYH-cis); (d) trans-cinnamate-intercalated LYH after 52.5 h of UV exposure of the suspension in isopropanol; Figure S2: absorption spectra of aliquots sampled at different time intervals during UV irradiation of trans-cinnamate-intercalated LYH suspension in isopropanol; Figure S3: Diffraction patterns of the initial trans-cinnamate-intercalated LYH and the product of its soaking in isopropanol for 48 h.

## References

[1] Pathem B.K., Claridge S.A., Zheng Y.B., Weiss P.S. (2013). Molecular switches and motors on surfaces. Annu. Rev. Phys. Chem..

[2] Baroncini M., Groppi J., Corra S., Silvi S., Credi A. (2019). Light-Responsive (Supra)Molecular Architectures: Recent Advances. Adv. Opt. Mater..

[3] Feringa B.L. (2001). In control of motion: From molecular switches to molecular motors. Acc. Chem. Res..

[4] Castiglioni F., Danowski W., Perego J., Leung F.K.C., Sozzani P., Bracco S., Wezenberg S.J., Comotti A., Feringa B.L. (2020). Modulation of porosity in a solid material enabled by bulk photoisomerization of an overcrowded alkene. Nat. Chem..

[5] Natali M., Giordani S. (2012). Molecular switches as photocontrollable “smart” receptors. Chem. Soc. Rev..

[6] Jan Van Der Molen S., Liljeroth P. (2010). Charge transport through molecular switches. J. Phys. Condens. Matter.

[7] Gust D., Moore T.A., Moore A.L. (2006). Molecular switches controlled by light. Chem. Commun..

[8] Li H., Martinez M.R., Perry Z., Zhou H.C., Falcaro P., Doblin C., Lim S., Hill A.J., Halstead B., Hill M.R. (2016). A Robust Metal–Organic Framework for Dynamic Light-Induced Swing Adsorption of Carbon Dioxide. Chem. Eur. J..

[9] Murakami H., Kawabuchi A., Kotoo K., Kunitake M., Nakashima N. (1997). A Light-Driven Molecular Shuttle Based on a Rotaxane. J. Am. Chem. Soc..

[10] Collin J.-P., Sauvage J.-P. (2005). Transition Metal-complexed Catenanes and Rotaxanes as Light-driven Molecular Machines Prototypes. Chem. Lett..

[11] Abellán G., Coronado E., Martí-Gastaldo C., Ribera A., Jordá J.L., García H. (2014). Photo-switching in a hybrid material made of magnetic layered double hydroxides intercalated with azobenzene molecules. Adv. Mater..

[12] Abellán G., Jordá J.L., Atienzar P., Varela M., Jaafar M., Gómez-Herrero J., Zamora F., Ribera A., García H., Coronado E. (2015). Stimuli-responsive hybrid materials: Breathing in magnetic layered double hydroxides induced by a thermoresponsive molecule. Chem. Sci..

[13] Li W., Yan D., Gao R., Lu J., Wei M., Duan X. (2013). Recent advances in stimuli-responsive photofunctional materials based on accommodation of chromophore into layered double hydroxide nanogallery. J. Nanomater..

[14] Yapryntsev A.D., Baranchikov A.E., Ivanov V.K. (2020). Layered rare-earth hydroxides: A new family of anion-exchangeable layered inorganic materials. Russ. Chem. Rev..

[15] Yan D., Wei M. (2015). Photofunctional Layered Materials.

[16] Strimaite M., Harman C.L.G., Duan H., Wang Y., Davies G.-L., Williams G.R. (2021). Layered terbium hydroxides for simultaneous drug delivery and imaging. Dalt. Trans..

[17] Lee B.-I., Lee E.-S., Byeon S.-H. (2012). Assembly of Layered Rare-Earth Hydroxide Nanosheets and SiO_2_ Nanoparticles to Fabricate Multifunctional Transparent Films Capable of Combinatorial Color Generation. Adv. Funct. Mater..

[18] Clampitt B.H., Callis J.W. (1962). Photochemical isomerization of cinnamic acid in aqueous solutions. J. Phys. Chem..

[19] Kim H., Gang B., Jung H., Byeon S.H. (2019). Cinnamate intercalated-layered yttrium hydroxide: A potential hybrid UV filter. J. Solid State Chem..

[20] Mohsin S.M.N., Hussein M.Z., Sarijo S.H., Fakurazi S., Arulselvan P., Hin T.-Y.Y. (2013). Synthesis of (cinnamate-zinc layered hydroxide) intercalation compound for sunscreen application. Chem. Cent. J..

[21] Li Y., Tang L., Ma X., Wang X., Zhou W., Bai D. (2017). Synthesis and characterization of Zn-Ti layered double hydroxide intercalated with cinnamic acid for cosmetic application. J. Phys. Chem. Solids.

[22] Timár Z., Varga G., Szabados M., Csankó K., Alapi T., Forano C., Prevot V., Sipos P., Pálinkó I. (2020). Structural insight into the photoinduced E→Z isomerisation of cinnamate embedded in ZnAl and MgAl layered double hydroxides. J. Mol. Struct..

[23] Zhao M., Gao M., Dai C., Zou C., Yang Z., Wu X., Liu Y., Wu Y., Fang S., Lv W. (2017). Investigation of Novel Triple-Responsive Wormlike Micelles. Langmuir.

[24] Saleh N., Bufaroosha M.S., Moussa Z., Bojesomo R., Al-Amodi H., Al-Ahdal A. (2020). Encapsulation of Cinnamic Acid by Cucurbit[7]uril for Enhancing Photoisomerization. Molecules.

[25] Valim J., Kariuki B.M., King J., Jones W. (1992). Photoactivity of cinnamate-intercalates of layered double hydroxides. Mol. Cryst. Liq. Cryst. Sci. Technol. Sect. A Mol. Cryst. Liq. Cryst..

[26] Kameshima Y., Nakada A., Isobe T., Nakajima A., Okada K. (2013). The effect of UV radiation on cinnamate/layered double hydroxide (LDH) composites. J. Ceram. Soc. Jpn..

[27] Feng P., Wang X., Zhao Y., Fang D.C., Yang X. (2018). Energy transfer between rare earths in layered rare-earth hydroxides. RSC Adv..

[28] Pereira C.C.L., Almeida M., Marçalo J., Monteiro B., Pereira L.C.J., Coutinho J.T., Coronado E., Baldoví J.J., Gaita-Ariño A. (2015). Magnetic Properties of the Layered Lanthanide Hydroxide Series Y_x_Dy_8−x_(OH)_20_Cl_4_·6H_2_O: From Single Ion Magnets to 2D and 3D Interaction Effects. Inorg. Chem..

[29] Abellán G., Espallargas G.M., Lorusso G., Evangelisti M., Coronado E. (2015). Layered gadolinium hydroxides for low-temperature magnetic cooling. Chem. Commun..

[30] Ogawa M., Kaiho H. (2002). Homogeneous precipitation of uniform hydrotalcite particles. Langmuir.

[31] Yapryntsev A., Abdusatorov B., Yakushev I., Svetogorov R., Gavrikov A., Rodina A., Fatyushina Y., Baranchikov A., Zubavichus Y., Ivanov V. (2019). Eu-Doped layered yttrium hydroxides sensitized by a series of benzenedicarboxylate and sulphobenzoate anions. Dalt. Trans..

[32] Carvalho Filho M.A.S., Fernandes N.S., Fertonani F.L., Ionashiro M. (2003). A thermal behaviour study of solid-state cinnamates of the latter trivalent lanthanides and yttrium(III). Thermochim. Acta.

[33] Moskalenko E., Sadovnikov A., Baranchikov A., Goldt A., Kozik V., Ivanov V. (2014). Synthesis of Nanocrystalline Titania via Microwave-Assisted Homogeneous Hydrolysis Under Hydrothermal Conditions. Curr. Microw. Chem..

[34] Yapryntsev A.D., Baranchikov A.E., Skogareva L.S., Goldt A.E., Stolyarov I.P., Ivanova O.S., Kozik V.V., Ivanov V.K. (2015). High-yield microwave synthesis of layered Y_2_(OH)_5_NO_3_·xH_2_O materials. CrystEngComm.

[35] Feng Z., Xiao D., Liu Z., Hou G., Xu J. (2021). “X Factor” in the Structure and Anion Exchange of Layered Yttrium Hydroxides. J. Phys. Chem. C.

[36] Geng F., Xin H., Matsushita Y., Ma R., Tanaka M., Izumi F., Iyi N., Sasaki T. (2008). New layered rare-earth hydroxides with anion-exchange properties. Chem. Eur. J..

[37] Liao L., Zhao N., Xia Z. (2012). Hydrothermal synthesis of Mg-Al layered double hydroxides (LDHs) from natural brucite and Al(OH)_3_. Mater. Res. Bull..

[38] Benito P., Guinea I., Labajos F.M., Rives V. (2008). Microwave-assisted reconstruction of Ni, Al hydrotalcite-like compounds. J. Solid State Chem..

[39] Zhao M.R., Qi Z.L., Chen F.X., Yue X.X. (2014). Kinetics of non-isothermal decomposition of cinnamic acid. Russ. J. Phys. Chem. A.

[40] Allen S.D.M., Almond M.J., Bruneel J.L., Gilbert A., Hollins P., Mascetti J. (2000). Photodimerization of trans-cinnamic acid and its derivatives: A study by vibrational microspectroscopy. Spectrochim. Acta Part A Mol. Biomol. Spectrosc..

[41] Yapryntsev A.D., Skogareva L.S., Gol’dt A.E., Baranchikov A.E., Ivanov V.K. (2015). Synthesis of a peroxo derivative of layered yttrium hydroxide. Russ. J. Inorg. Chem..

[42] Li Z.J., Cai L., Mei R.F., Dong J.W., Li S.Q., Yang X.Q., Zhou H., Yin T.P., Ding Z.T. (2015). A highly efficient transformation of cis- to trans-cinnamic acid derivatives by iodine. Tetrahedron Lett..

[43] Salum M.L., Erra-Balsells R. (2013). High purity cis-cinnamic acid preparation for studying physiological role of trans-cinnamic and cis-cinnamic acids in higher plants. Environ. Control Biol..

[44] Hanai K., Kuwae A., Takai T., Senda H. (2001). A comparative vibrational and NMR study of cis-cinnamic acid polymorphs and trans-cinnamic acid. Spectrochim. Acta Part A Mol. Biomol. Spectrosc..

[45] Hindocha S.A., McIntyre L.J., Fogg A.M. (2009). Precipitation synthesis of lanthanide hydroxynitrate anion exchange materials, Ln_2_(OH)_5_NO_3_·H_2_O (Ln = Y, Eu–Er). J. Solid State Chem..

[46] Nakagawa I., Walter J.L. (1969). Optically active crystal vibrations of the alkali-metal nitrates. J. Chem. Phys..

[47] Socrates G. (2001). IR and Raman Characteristic Group Frequencies: Tables and Charts.

[48] Nakamoto K. (2009). Infrared and Raman Spectra of Inorganic and Coordination Compounds: Part A.

[49] Mcintyre L.J., Jackson L.K., Fogg A.M. (2008). Ln_2_(OH)_5_NO_3_·xH_2_O (Ln = Y, Gd-Lu): A Novel Family of Anion Exchange Intercalation Hosts. Chem. Mater..

[50] Fogg A.M., Williams G.R., Chester R., O’Hare D. (2004). A novel family of layered double hydroxides—[MAl_4_(OH)_12_](NO_3_)_2_·xH_2_O (M = Co, Ni, Cu, Zn). J. Mater. Chem..

[51] Newman S.P., Jones W. (1999). Comparative Study of Some Layered Hydroxide Salts Containing Exchangeable Interlayer Anions. J. Solid State Chem..

[52] Xu M., Wei M. (2018). Layered Double Hydroxide-Based Catalysts: Recent Advances in Preparation, Structure, and Applications. Adv. Funct. Mater..

[53] Song L., Shi W., Lu C. (2016). Confinement effect in layered double hydroxide nanoreactor: Improved optical sensing selectivity. Anal. Chem..

[54] Gu Q., Su F., Ma S., Sun G., Yang X. (2015). Controllable luminescence of layered rare-earth hydroxide composites with a fluorescent molecule: Blue emission by delamination in formamide. Chem. Commun..

[55] Gu Q., Su F., Ma L., Ma S., Sun G., Yang X. (2015). Intercalation of coumaric acids into layered rare-earth hydroxides: Controllable structure and photoluminescence properties. J. Mater. Chem. C.

[56] Shi W., Lin Y., Zhang S., Tian R., Liang R., Wei M., Evans D.G., Duan X. (2013). Study on UV-shielding mechanism of layered double hydroxide materials. Phys. Chem. Chem. Phys..

[57] Adam N., Mohd Ghazali S.A.I.S., Dzulkifli N.N., Hak C.R.C., Sarijo S.H. (2019). Intercalations and characterization of zinc/aluminium layered double hydroxide-cinnamic acid. Bull. Chem. React. Eng. Catal..

