# Supplementary material for: Cinnamate-Intercalated Layered Yttrium Hydroxide: UV Light-Responsive Switchable Material
Source: Micromachines (Basel). 2023 Sep 19;14(9):1791. doi: 10.3390/mi14091791 (PMC10534862; doi:10.3390/mi14091791)
Supplement: Supplementary file 1 [file micromachines-14-01791-s001.zip › micromachines-2561635-supplementary.pdf]

Electronic Supporting Information

Cinnamate-Intercalated Layered Yttrium Hydroxide: UV Light-Responsive Switchable Material

Maria A. Teplonogova, Alexey D. Yapryntsev, Alexander E. Baranchikov, Vladimir K. Ivanov*

Kurnakov Institute of General and Inorganic Chemistry of the Russian Academy of Sciences, Leninsky pr. 31, Moscow, 119991, Russia.

E-mail: van@igic.ras.ru

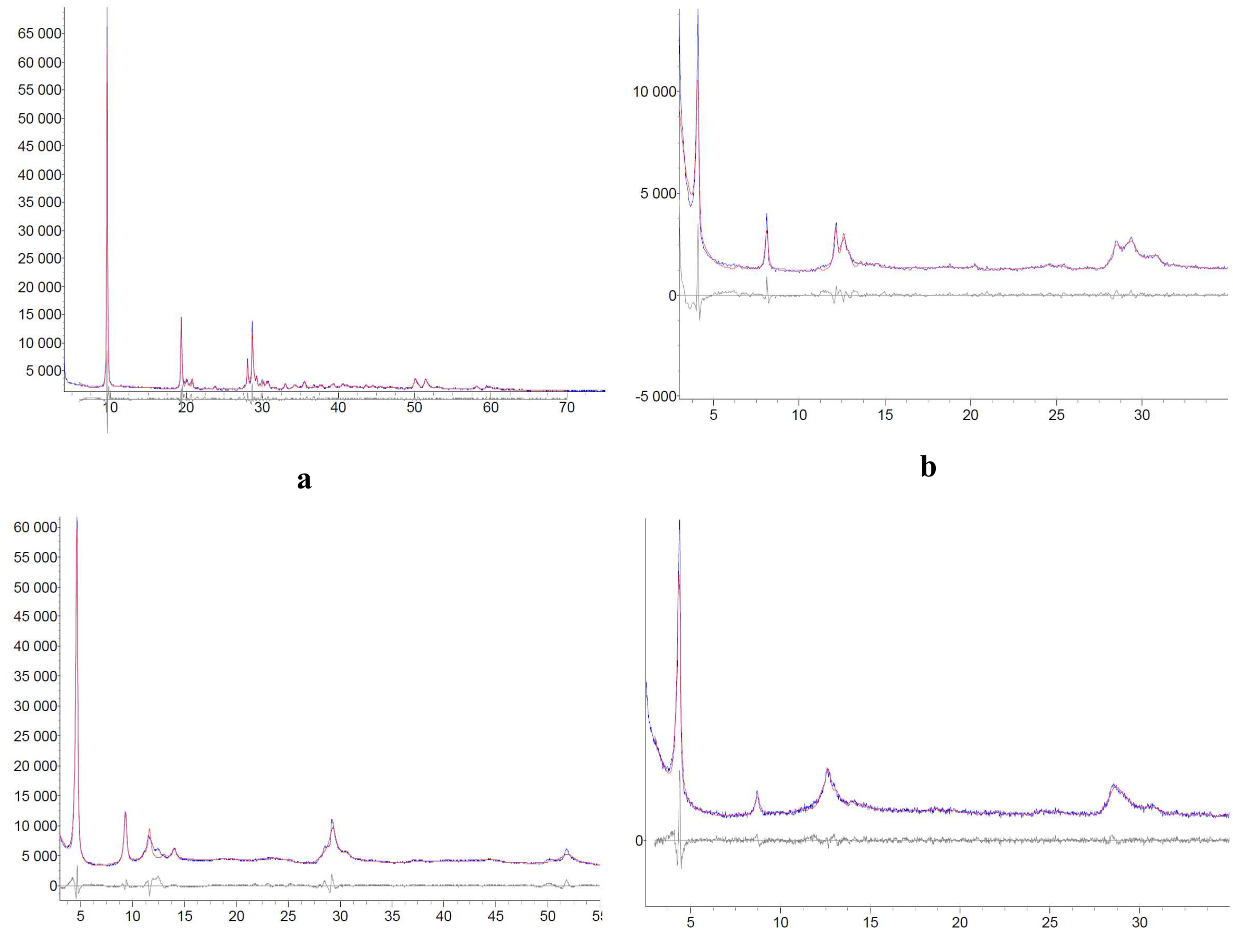

a b c d

Figure S1. The full-profile refinement charts for the samples of (a) LYH-NO_3; (b) trans-cinnamate intercalated LYH (LYH-trans); (c) cis-cinnamate intercalated LYH (LYH-cis); (d) trans-cinnamate intercalated LYH after 52.5 h of UV-exposure of the suspension in isopropanol.

Table S1. The results of full-profile refinement for LYH-NO_3, trans-cinnamate (LYH-trans), and cis-cinnamate (LYH-cis) intercalated LYH sample (space group $P2_1$).

Sample	a, Å	b, Å	c, Å	β, °	Rwp
LYH-NO_3	13.2290(13)	7.0100(20)	9.1620(16)	87.6550(93)	7.86
LYH-trans	13.955(28)	7.485(10)	21.712(12)	81.29(13)	8.12
LYH-cis	13.618(15)	6.972(11)	19.001(11)	87.084(50)	5.05
LYH-trans after 52.5 h of UV-exposure	14.474(21)	6.254(12)	20.640(31)	84.518(44)	6.77

Table S2. Position of the bands (cm^{-1}) in the Raman spectra of layered yttrium hydroxide (LYH) intercalated with cinnamate and assignment of the vibrations according to literature data [1]–[4].

Layered yttrium hydroxo-nitrate	LYH intercalated with trans-cinnamate at 120°C	LYH intercalated with cis-cinnamate at 120°C	LYH intercalated with trans-cinnamate at 160°C	Cinnamic acid [3]		Vibration
				monomer	dimer	
	621	621	620	621	618	ring deformation
719						δ(NO_3^-) [2]
	744				768	ring (aliphatic)
					808	C-COOH
	853	851	854	846		
	876	883	876	873		
	1002	1002	1002	1000	1000	ring
	1029	1031	1029	1026	1032	C-H in plane
1055						ν(NO_3^-), δ(OH) [2]
	1158	1157	1158	1161	1156	C-H in plane; inorganic CO_3^{2-} [1]
					1169	C-H in plane
	1182	1180	1183	1179	1187	C-H in plane [3] / cinnamate [1] / C-O in plane [4]
	1203	1203	1204	1210	1207	
					1215	
	1253	1253	1253	1258		
					1289	C-H saturated
					1360	
1406			1395			
	1434		1435			O-H in plane [4]
	1456	1451	1457	1450		C-H unsaturated; inorganic CO_3^{2-} [1]
	1497	1495	1497	1494		C-H unsaturated
					1586	C=C (aromatic)
	1603	1600	1603	1599	1599	
	1644	1642	1644	1637		C=C (isolated) [1] C=C (aliphatic) [3]
					2915	C-H saturated
					2942	
					2964	
					2986	
	3060	3060	3060		3058	O-H[1]; C-H unsaturated [3]

Table S3. Position of the bands (cm^{-1}) in the infrared spectra of layered yttrium hydroxide (LYH) intercalated with cinnamate and assignment of the vibrations according to literature data [5].

Trans-cinnamic acid	LYH intercalated with trans-cinnamate	LYH intercalated with cis-cinnamate	Mg_2Al-LDH intercalated with trans-cinnamate [5]	Mg_2Al-LDH intercalated with cis-cinnamate [5]	Vibration [5]
1667					
1626	1641	1636	1637	1637	n(C=C)cinn
1599					
1576	1578	1577	1576		n(CC)ar + b(CH) + a(CCC)
		1555		1550	$n_{as}(COO^-)$
	1545		1537		
1494	1495	1497	1499	1493	b(CH) + n(CC)ar
1447	1452	1448			b(CH) + n(CC)ar
		1437			
1416	1423	1420			O-H
	1398	1399	1394		$n_s(COO^-)$
		1356		1363	
1333, 1311					
1283	1292	1292	1290		b(CH)cinn + n(CC)
	1250	1249	1248		b(CH)cinn
1219				1211	
1205	1203	1203			
1175				1184	b(CH)ar + n(CC)ar
1158		1149			
1098	1070	1072	1070	1076	b(CH)ar + n(CC)ar
	1047	1045			
1027	1028	1028		1032	b(CH)ar + n(CC)ar
1000				1007	a(CCC) + n(CCC)
976	974	978	978	968	g(CCH)cinn
942				941	
909		917			
878	876	878	880	882	g(CH)ar
844	850	848	850	856	b_s(COO-) + b(CH)cinn
765	771	775	777	764	g(CH)ar + g(HCCO) + g(OCOC)
	737				
707	715	719	717		F(CCC) + g(CH)ar

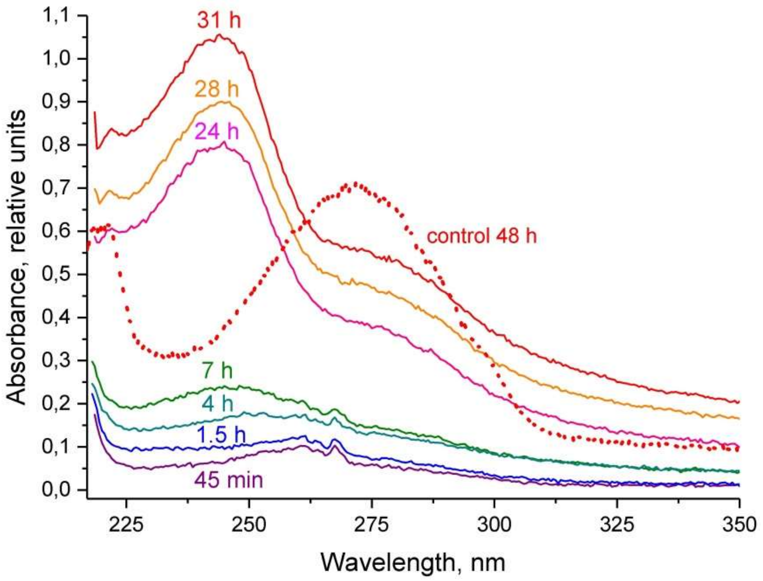

Figure S2. Absorption spectra of aliquots sampled at different time intervals during UV-irradiation of trans-cinnamate intercalated LYH suspension in isopropanol.

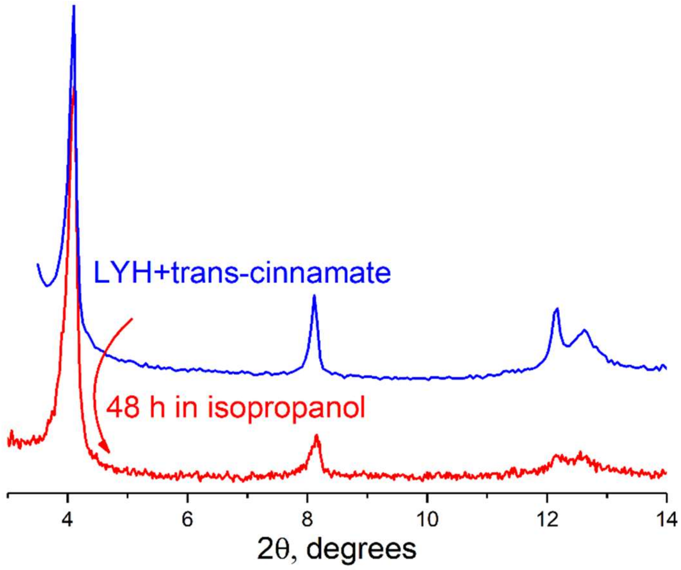

Figure S3. Diffraction patterns of the initial trans-cinnamate intercalated LYH and the product of its soaking in isopropanol for 48 h.

References

[1] Socrates, G. *IR and Raman characteristic group frequencies. Tables and charts*. 2001

[2] Yapryntsev, A. D., Skogareva, L. S., Gol'dt, A. E., Baranchikov, A. E., Ivanov, V. K. *Synthesis of a peroxo derivative of layered yttrium hydroxide* // **Russian Journal of Inorganic Chemistry**, 2015, V. 60, № 9, P. 1027–1033, DOI: 10.1134/S0036023615090211

[3] Allen, S. D. M., Almond, M. J., Bruneel, J. L., Gilbert, A., Hollins, P., Mascetti, J. *Photodimerization of trans-cinnamic acid and its derivatives: A study by vibrational*

microspectroscopy // **Spectrochimica Acta - Part A: Molecular and Biomolecular Spectroscopy**, 2000, V. 56, № 12, P. 2423–2430, DOI: 10.1016/S1386-1425(00)00294-8

[4] Hanai, K., Kuwae, A., Takai, T., Senda, H. *A comparative vibrational and NMR study of cis-cinnamic acid polymorphs and trans-cinnamic acid* // 2001, V. 57, P. 513–519

[5] Timár, Z., Varga, G., Szabados, M., Csankó, K., Alapi, T., Forano, C., *et al. Structural insight into the photoinduced E→Z isomerisation of cinnamate embedded in ZnAl and MgAl layered double hydroxides* // **Journal of Molecular Structure**, 2020, V. 1219, DOI: 10.1016/j.molstruc.2020.128561
